# Variety of Alternative Stable Phase-Locking in Networks of Electrically Coupled Relaxation Oscillators

**DOI:** 10.1371/journal.pone.0086572

**Published:** 2014-02-10

**Authors:** Pierre Meyrand, Tiaza Bem

**Affiliations:** 1 Univ. Bordeaux, IMN, UMR 5293, Bordeaux, France; 2 CNRS, IMN, UMR 5293, Bordeaux, France; 3 Nalecz Institute of Biocybernetics and Biomedical Engineering, Polish Academy of Sciences, Warsaw, Poland; Northwestern University, United States of America

## Abstract

We studied the dynamics of a large-scale model network comprised of oscillating electrically coupled neurons. Cells are modeled as relaxation oscillators with short duty cycle, so they can be considered either as models of pacemaker cells, spiking cells with fast regenerative and slow recovery variables or firing rate models of excitatory cells with synaptic depression or cellular adaptation.

It was already shown that electrically coupled relaxation oscillators exhibit not only synchrony but also anti-phase behavior if electrical coupling is weak. We show that a much wider spectrum of spatiotemporal patterns of activity can emerge in a network of electrically coupled cells as a result of switching from synchrony, produced by short external signals of different spatial profiles. The variety of patterns increases with decreasing rate of neuronal firing (or duty cycle) and with decreasing strength of electrical coupling. We study also the effect of network topology - from all-to-all – to pure ring connectivity, where only the closest neighbors are coupled.

We show that the ring topology promotes anti-phase behavior as compared to all-to-all coupling. It also gives rise to a hierarchical organization of activity: during each of the main phases of a given pattern cells fire in a particular sequence determined by the local connectivity. We have analyzed the behavior of the network using geometric phase plane methods and we give heuristic explanations of our findings.

Our results show that complex spatiotemporal activity patterns can emerge due to the action of stochastic or sensory stimuli in neural networks without chemical synapses, where each cell is equally coupled to others via gap junctions. This suggests that in developing nervous systems where only electrical coupling is present such a mechanism can lead to the establishment of proto-networks generating premature multiphase oscillations whereas the subsequent emergence of chemical synapses would later stabilize generated patterns.

## Introduction

Electrical synapses have been shown to be important in the regulation of neuronal and glial cell activity in developing, adult and injured central nervous system (CNS) [1]–[5]. Electrical coupling between cells is mediated by intercellular channels that enable cell-to-cell electrical communication as well as intercellular transport of small molecules. Whereas in vertebrates these channels are formed by a large family of hemi-channels called connexins [6]–[7], homologous molecules have been found in invertebrates where the gap junction protein is called innexin [8]–[9]. In both invertebrate and vertebrate systems gap junctions undergo regulation of their expression and conductance via different mechanisms varying from neuromodulation to transcriptional regulation [10]–[13] including activity dependent mechanisms [14]. For example, in adult systems, the strength of gap junction coupling can be modified by many agents such as nitric oxide via cGMP [15] or dopamine [16]–[17]. In the developing nervous system the expression of connexins increases during the first postnatal weeks in the cortex and then decreases [18]–[19] whereas in the spinal cord similar changes occur mainly during late embryonic and late postnatal life [18], [20]–[21].

Gap junctions play an important role in the CNS physiology. The most obvious is their ability to equalize the membrane potentials of cells and therefore to create clusters of cells expressing similar electrical activity. However, using a modeling approach it has been shown that electrically coupled neurons can also express an anti-synchronous behavior. Indeed, both in network models comprised of relaxation oscillators of sufficiently small duty cycle (i.e., small spike duration compared to the duration of the cycle) [22]–[23] or in networks composed of integrate-and-fire units [24]–[26] weak electrical coupling may lead, although via different mechanisms, to anti-synchrony (see also Wang-Buzsaki model neurons in [27]). Importantly, all these models show the capacity of electrically coupled neurons to generate only two behaviors: synchrony (in-phase locking, IP) or anti-synchrony (anti-phase locking, AP). However, in biological systems, in early development where chemical synapses are not yet fully established and only electrical synapses are present, it is not clear what factors contribute to the ability of embryonic circuits to generate their first patterned activity. Therefore the question arises as to what extent electrical coupling contributes to the generation of activity patterns that are more complex than simple synchrony or anti-synchrony.

In this paper we show that a large-scale neural network comprised of relaxation oscillators interconnected solely by electrical synapses expresses a much wider spectrum of multiphase patterns. A relaxation oscillator is a model commonly used to describe a cellular pacemaker (slow envelope of membrane potential in bursting neurons) and in the case of a short duty cycle, when the duration of the active phase is a negligible fraction of the oscillatory period (1–2%), is also applicable for spiking neurons, in which the intrinsic regenerative mechanism is fast compared to the recovery variable [28], [29]. Moreover, this model is analogous to firing-rate models of excitatory neural networks with slow negative feedback like synaptic depression or cellular adaptation, in which neurons do not exhibit, by themselves, pacemaker properties, like for example in developing CPG networks of the spinal cord [30].

In this study we looked for a full spectrum of stable multiphase solutions which coexist with the IP solution (i.e. synchrony) in the network comprised of 24 or 60 electrically coupled oscillators. We obtained various multiphase spatiotemporal patterns by applying transient switching stimuli of different spatial profiles distributed among cells expressing synchronous behavior. The network is organized in a ring-like structure parameterized by the number of connections of an individual cell to its closest neighbors, so we can gradually change the network topology from a pure ring, with only two connections per cell, up to all-to-all coupling. Such a ring-like organization of network architecture was introduced in order to control the extent of local coupling since in biological networks electrical synapses are made between membranes of neighboring cells. Our goal was to understand how the robustness of a given pattern depends on the number of phases it contains, the number of cells firing in each of the phases, on the network topology and on the cells' duty cycle. We first found a domain of parameters, namely the coupling strength and connectivity, in which a given pattern is stable in the presence of noise of different amplitudes. Here the robustness of the pattern decreased with the increasing number of phases involved but was weakly dependent on the network architecture, except for the AP pattern. We also showed a subtle structure of multiphase patterns in networks with a small number of connections per cell. Here subpopulations of cells which are active during each of the main phases of a pattern do not fire synchronously but in a given order thereby creating a sub-pattern of activity.

In parallel, using geometrical phase plane analysis, we showed that deflections of the phase plane trajectory occurring during the evolution in the silent phase due to interaction with active cells are critically important for the existence of a given solution. Moreover, we described a relation between the number of phases in a solution and the robustness of the pattern and how this changes as a function of network architectures. We also showed why decreasing the cell's duty cycle allows for the existence of solutions of increased number of phases. Finally, we analyzed the stability of asymmetrical 2-phase patterns, with unequal number of cells firing in each of the two phases. We demonstrated that in networks with a small number of connections per cell highly asymmetrical patterns cannot be generated.

## Results

### Network connectivity pattern

**Figure 1:**
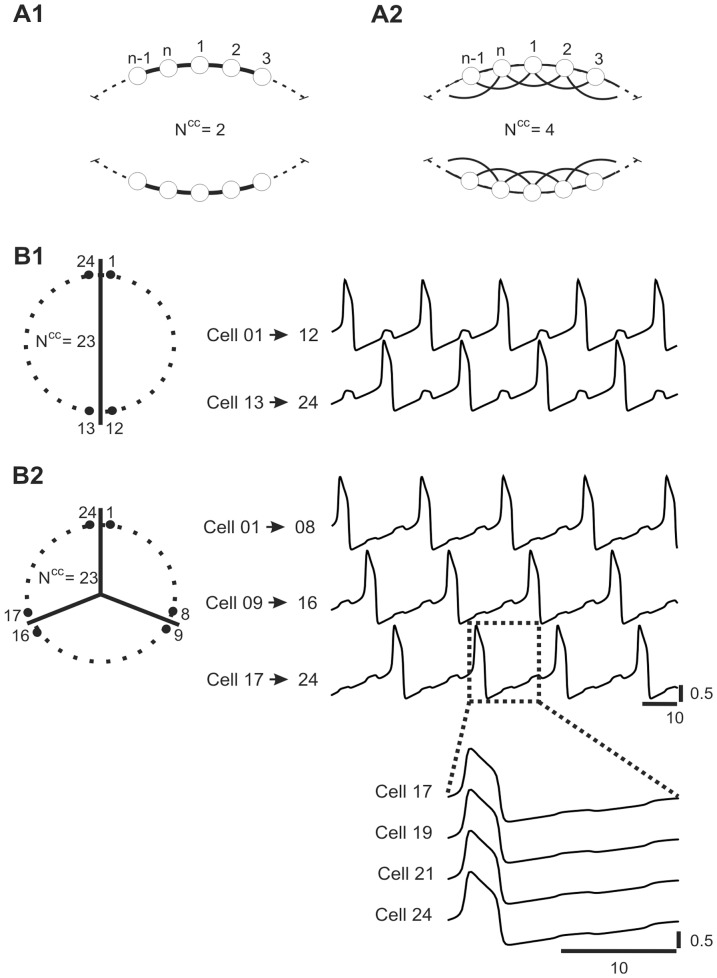
Multiple activity patterns expressed by network of electrically coupled oscillatory cells. A. Examples of wiring diagrams of network connectivity for different numbers of closest connected cells N^cc^. B. 2-phase and 3-phase patterns expressed by fully connected network. Equally numerous groups of cells (see scheme of cells contribution to different groups, left) express activity in 2 (B1) or 3 (B2) different phases of oscillatory cycle. Note that cells belonging to a given group fire synchronously (see insert, B2). Parameters: N = 24, N^cc^ = 23, g^el^ = 0.12 (B).

We considered a ring-like network model consisting of N cells, in which each cell was connected electrically to a given number of neighboring cells (Number of connected cells N^cc^) varying from 2 to N-1, so network connectivity could be gradually changed from a closest neighbor pattern (N^cc^ = 2) (which we call “N^cc^2” network or “ring network”) to an “all to all” coupling pattern (N^cc^ = N-1) (Fig. 1A1). For example, in Figure 1A2 N^cc^ is equal to 4. While changing the connectivity parameter (N^cc^) the total electrical conductance per single cell was kept constant.

### Multiple activity patterns coexist with synchrony

Starting from in-phase (IP) oscillations and by stimulating different groups of cells with a transient stimulus (see I^in^, equation 1) we were able to find a number of stable activity patterns which were co-existing with the IP solution. Thereafter the stability of these solutions was tested as a function of the electrical coupling strength (g^el^) for different connectivities (N^cc^) and different levels of noise (σ_noise).

**Figure 2:**
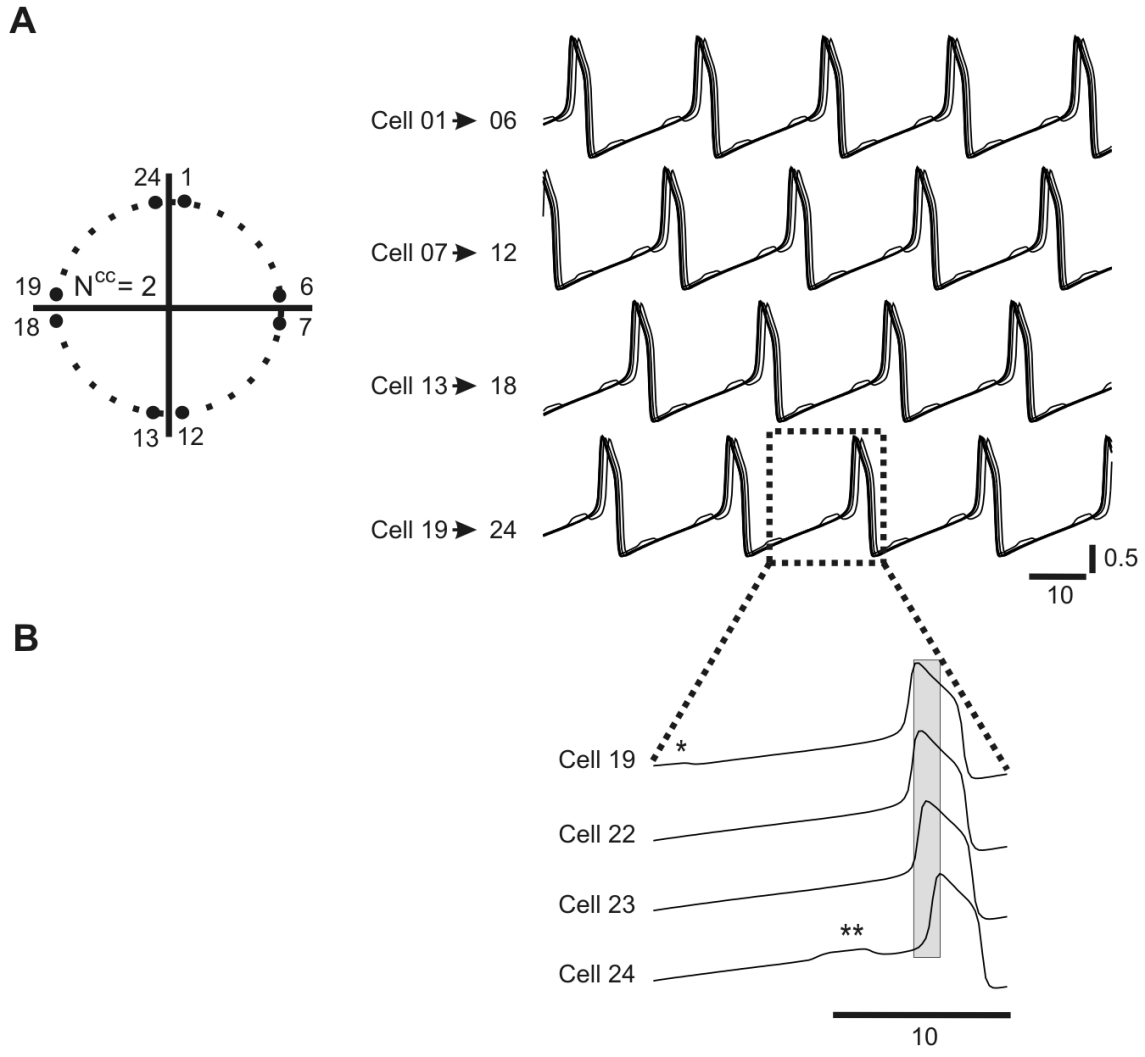
4-phase activity pattern. Ring network (N^cc^ = 2) is divided into 4 topologically compact groups of cells (left) which fire in 4 distinct phases (right). Whereas behaviors among the groups are identical cells within each group have different voltage trajectories (stars indicate visually observed differences, insert) and do not fire synchronously (see voltage trajectories within rectangle, insert). Parameters: N = 24, N^cc^ = 2, g^el^ = 0.08.

We first tested the network with all-to-all connectivity (N^cc^ = 23) consisting of 24 relaxation oscillators by stimulating a group containing 12 neighboring cells (Fig. 1B1) or groups containing 8 neighboring cells (Fig. 1B2). Following such stimulation we obtained either a 2-phase solution in which the two groups of cells oscillating in anti-phase (AP) (right panel, Fig. 1B1) or a 3-phase solution, with a phase shift Ф between the groups equal to 1/3 (right panel, Fig. 1B2). Importantly, cells belonging to the same group oscillated in pure synchrony (see cells 17–24, bottom panel, Fig. 1 B2). It must be noted that under all-to-all coupling (N^cc^ = 23) it was not possible to switch from the IP to the 4-phase solution. However, such a solution was easily obtained if the connectivity pattern was changed to N^cc^2 connectivity, as illustrated in Figure 2. In such a network four groups of cells were oscillating with a phase shift Ф equal to approximately 1/4 but, by contrast to all-to-all coupling, pure synchrony of cells within each group was not present (see slight phase shift (grey bar) between cell 19–24, bottom panel, Fig. 2). Moreover, whereas in the fully coupled network within a given group of cells voltage traces were identical (bottom panel, Fig. 1B2), here they differ (compare cell 24 with other cells, see stars, bottom panel, Fig. 2).

### Patterns' stability as a function of network connectivity

**Figure 3:**
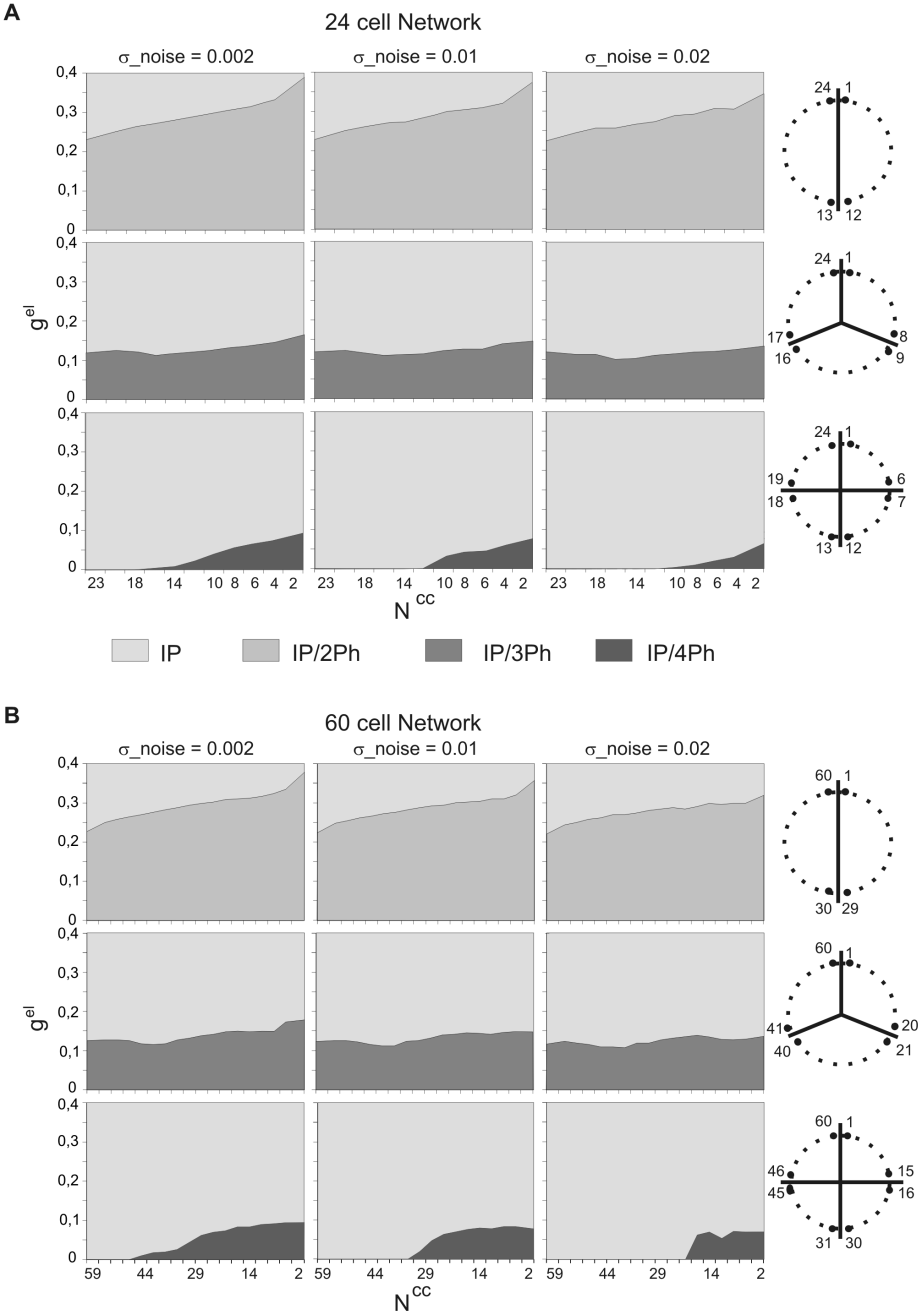
Occurrence of activity patterns as a function of electrical conductance and network connectivity. Shown are domains of stability of 2, 3 and 4-phase solutions in (g^el^, N^cc^) parameter space in 24 (A) and 60 cell network (B). Note that with increasing the number of groups expressing different phases (see schemes of groups, right) the robustness of the solution diminished (left). Parameters: σ_noise indicates standard deviation of independent identically Gaussian distributed random current values for each 0.2 unit integration step in A and B.

In the next step we searched for a domain of coupling strength and connectivity parameters in which 2, 3 and 4-phase solutions were stable (and co-existing with IP). As illustrated in Figure 3A, the 2-phase solution (i.e. AP oscillations) (top row, dark grey) was found to be stable over a large range of electrical coupling strength, until g^el^ equal to 0.22–0.37, depending on the N^cc^, whereas above this limit only the IP solution was stable (top row, light grey areas). Interestingly, the robustness of the AP solution increased as a function of a decrease in the number of synapses per cell (see enlargement of the domains of stability from N^cc^ = 23 to N^cc^ = 2). The 3-phase solution was stable over a much narrower range of electrical coupling than the 2-phase solution and its robustness was weakly dependent on the network topology (middle row, dark gray areas). Finally, the 4-phase solution was stable only for a very small range of the coupling strength and moreover only for small number of synapses per cell (N^cc^<14, depending on the amplitude of noise) (bottom row, dark gray areas). These features of the stability of the three solutions were fairly resistant to increasing of the amplitude of noise (compare columns from left to right) as well as to increasing the number of cells in the network (compare Fig. 3A and 3B).

### Phase plane trajectory of the AP solution

**Figure 4:**
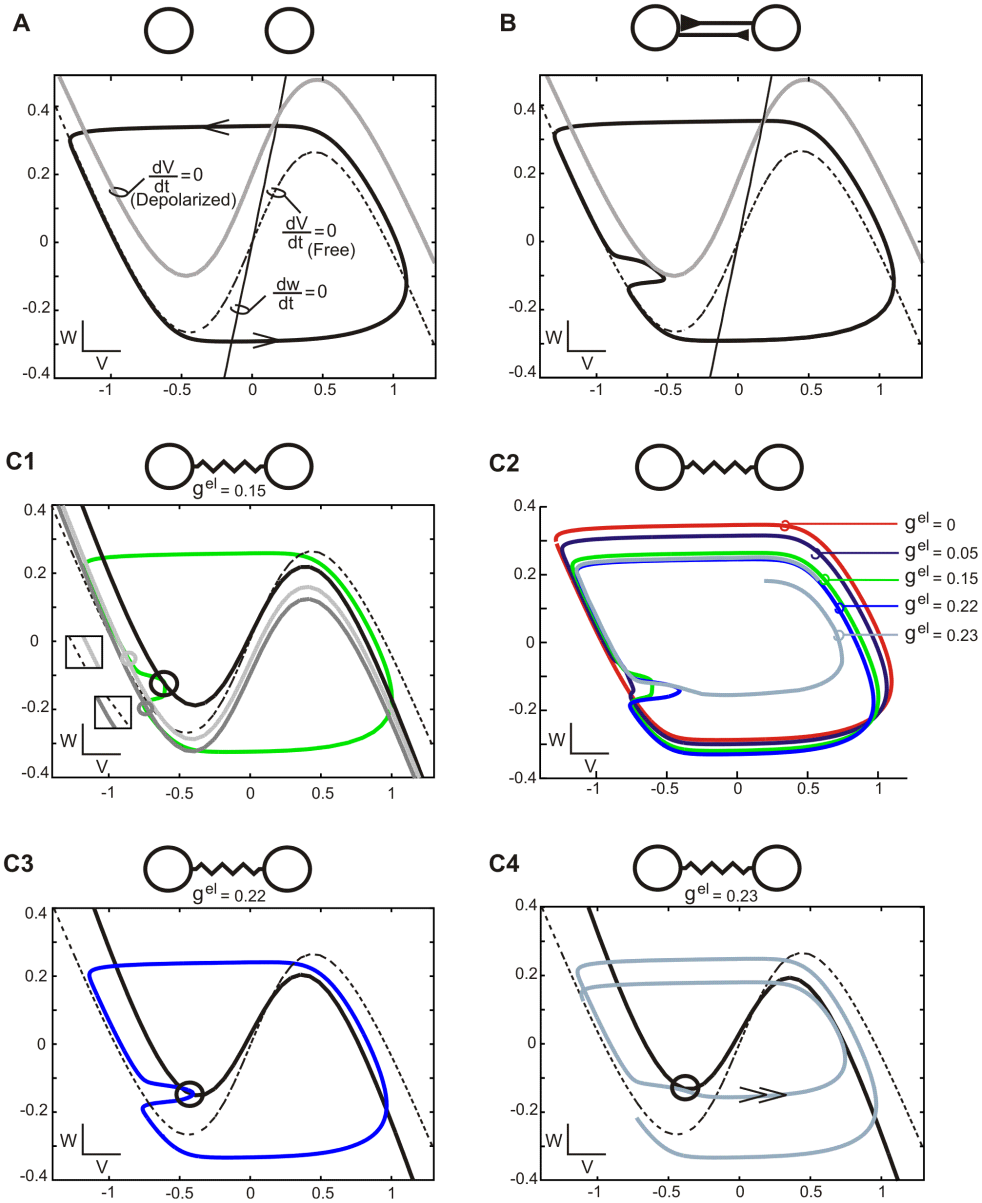
Phase plane evolution of oscillators involved in the 2-phase behavior in fully connected network. A. Trajectory of an uncoupled oscillatory cell (black curve) on the phase plane. Evolution along left and right branches of *V*-nullcline (dashed cubic curve) corresponds to the active and silent phases, respectively. Transitions between the phases occur when the cell jumps up from the left knee or jumps down from the right knee of the *V*-nullcline (see arrows). The speed of evolution is high during jumps as compared to evolution along *V*-nullcline when it is inversely proportional to the distance between the actual position of the cell and the *W*-nullcline (black straight line). Also shown is the *V*-nullcline for depolarized system (grey cubic curve). B. AP trajectory of 2 cells coupled reciprocally by excitatory synapses. The trajectory (black curve) is identical as in A, except for a right-sided deflection, corresponding to the evolution along the new *V*-nullcline (grey cubic curve) of the depolarized cell, which is shifted up with respect to the free nullcline (dashed cubic curve). C. AP trajectory of electrically coupled cells. The trajectory of two identical groups of cells expressing anti-phase oscillation (green curve, C1) is compressed comparing to the free cell trajectory (black curve, A) due to continues interaction of two groups all over the cycle. Shown are positions of cells before, during and after the other group's active phase (light grey, black and dark grey circle, respectively) and corresponding nullclines (light gray, black and dark grey cubic curves). Notice shift of the nullclines with respect to the free nullcline (dashed cubic curve) (inserts, C1). Increasing g^el^ produces gradual compression of the AP trajectory (color curves, C2) and changes a position of cells (black circles) with respect to the knee of *V*-nullcline (black cubic curve) during a maximal deflection (C3-4, see also C1). When the knee of the nullcline is shifted above a cells' actual position (see black circle and solid cubic curve) a jump up occurs directly from the deflection phase to the active phase (see double arrow) and the AP solution disappears (light blue curve) (C4). Parameters: N = 2 (A-B), 24 (C), N^cc^ = 23 (C), in B parameters as described in Methods. Phase plane coordinates: *V* (abscissa), *W* (ordinate). Trajectories and nullclines were calculated using the software XPPAUT developed by B. Ermentrout (http://www.pitt.edu/~phase/).

In order to understand why decreasing the number of synapses per single cell (N^cc^ low) increases the robustness of the AP solution (see Fig. 3A, top row) we first considered the phase plane trajectory of a free relaxation oscillator. (In all figures trajectories are presented on the phase plane with coordinates: *V* (abscissa) and *W* (ordinate)). Figure 4A shows a cubic curve called *V*-nullcline (dashed curve) where *dV/dt = 0*. The nullcline is calculated by setting the right side of equation [1] equal zero. This is the nullcline of a free cell, with the values of the synaptic current *I^el^* and the external current *I^in^* equal to zero. Another example of *V*-nullcline corresponds to the case when the free cell receives a depolarizing current (see *I^syn^*, equation [5]): here the *V*- nullcline (grey cubic curve) is shifted above the free nullcline. Notice that the larger the value of the current, the larger will be the distance between the free and shifted nullcline and also, that a hyperpolarizing current will produce a shift below the free nullcline (not shown). Finally, *W*-nullcline (black straight line) shows where *dW/dt = 0* (see equation [2]). We assume relaxation regime so *W* is very slow compared to *V*.

The free cell trajectory (black curve) consists of four pieces; two of them lie along the left and right branches of the *V*-nullcline and correspond to cell's silent and active phases, respectively, whereas other two pieces which begin at the knees of the nullcline correspond to jumping between these two phases (arrows, Fig. 4A). The jumping phases are very fast compared with the silent and active phases. The speed of evolution along the left and right branches is not constant but depends on the vertical distance between the cell's actual position on the phase plane and the *W*-nullcline. Moreover, in our model the speed of the evolution is higher during the active than during the silent phase due to the smaller time constant (τ_w_) (see equation [4]) which determines the speed of approaching the *W*- nullcline and therefore assures that the cell duty cycle (the proportion of the duration of the active phase to cycle duration) is short. Figure 4B illustrates the phase plane evolution of two such cells with short duty cycle generating an AP pattern when coupled by reciprocal excitation (black solid curve). When one of the cells jumps up from the silent to active phase (see bottom arrow) it crosses the voltage threshold for transmitter release (not shown) and therefore the partner cell is removed from the free cell trajectory due to a depolarizing synaptic current and evolves along the shifted *V*-nullcline (see grey curve, Fig. 4B). After the cell jumps down (onset of the silent phase) the partner cell is released from excitation and returns to the evolution along left branch of the free nullcline (dashed curve, Fig. 4B).

In contrast to the two examples described above, the trajectory of the two cells coupled with electrical synapses is different from the free cell trajectory over the entire cycle (Fig. 4C green curve). Indeed, here the cell is continuously coupled with its partner either in the active (as in Fig. 4B) or in the silent phase due to the electrical coupling. Here the value of the coupling current *I^el^* (see equation [3]) depends on the actual position of the two cells on the phase plane (i.e., on the difference of *V*-coordinates) which in turn influences the shape and the position of the cell's *V*-nullcline at any moment of the cycle. For example, in Fig 4C1 we consider a cell (upper light grey circle) just before its partner cell jumps up (not shown). Here the cell's nullcline (light grey curve) is shifted up with respect to the free cell nullcline (dashed curve) (see also upper insert). This is due to a depolarizing coupling current resulting from the difference in membrane potential of the two cells. Later in the cycle the cell reaches another position (middle black circle) in which *V*-nullcline is at its uppermost position (black solid curve) due to a strong depolarizing coupling current from the partner cell which is now in the active phase evolving along the right branch of the nullcline (not shown). Thereafter, when the partner cell jumps down (not shown) the cell (dark grey circle) is now ahead of its partner in the silent phase and with a higher *V*. This produces a hyperpolarizing current and a shift down of the cell's nullcline with respect to the free nullcline (cf. dark grey curve and dashed curve, respectively, see also lower insert). Thus, as a result of electrical coupling the *V*-nullclines as well as the cell trajectory become shrunken compared to the free cell nullcline and trajectory (compare cells nullclines and trajectory in Fig. 4A and C1). The deformation of the trajectory depends on the value of electrical coupling. Indeed, as illustrated in Fig. 4C2 the trajectory is less compressed for weaker coupling (g^el^ = 0.05) than for the stronger coupling (g^el^ = 0.22). Moreover, with a further increase of the coupling strength the trajectory of cells expressing AP behavior becomes significantly altered (g^el^ = 0.23, see grey curve) and this solution disappears.

### Existence of the AP solution

In order to understand why AP solution disappears at a given value of g^el^ we analyzed the cell position with respect to the knee of the *V*-nullcline (see black circles and black curves, Fig. 4 C1, C3-C4) at the moment of the cells higher depolarization at the silent phase Whereas for g^el^ = 0.15 the cell is still well above the knee of the nullcline (black circle, Fig. 4C1) for g^el^ = 0.22 the cell is already at the level of the knee and with a larger *V* due to a shift up of the nullcline resulting from the increase of the coupling current (black circle, Fig. 4C3). With a further slight increase of the coupling (g^el^ = 0.23) the nullcline is again slightly shifted up and therefore the cell trajectory now becomes situated below the knee (black circle, Fig. 4C4). As a result, instead of continuing evolution along the left branch of the nullcline the cell is jumping directly toward the right branch (double arrow) approaching its partner cell in the active phase and after a few cycles the initial AP solution is replaced by the IP solution (not shown).

In conclusion, we can see that a deflection of the cell trajectory occurring along the left branch of the nullcline, corresponding to the cell depolarization during the silent phase via the coupling with the active partner, is a critical moment of the evolution in the phase plane. Here the AP solution has to be lost if the coupling becomes too strong. For the purpose of the further analysis we will call this part of the trajectory a “critical deflection.”

### Size of the critical deflection is affected by the network connectivity

**Figure 5:**
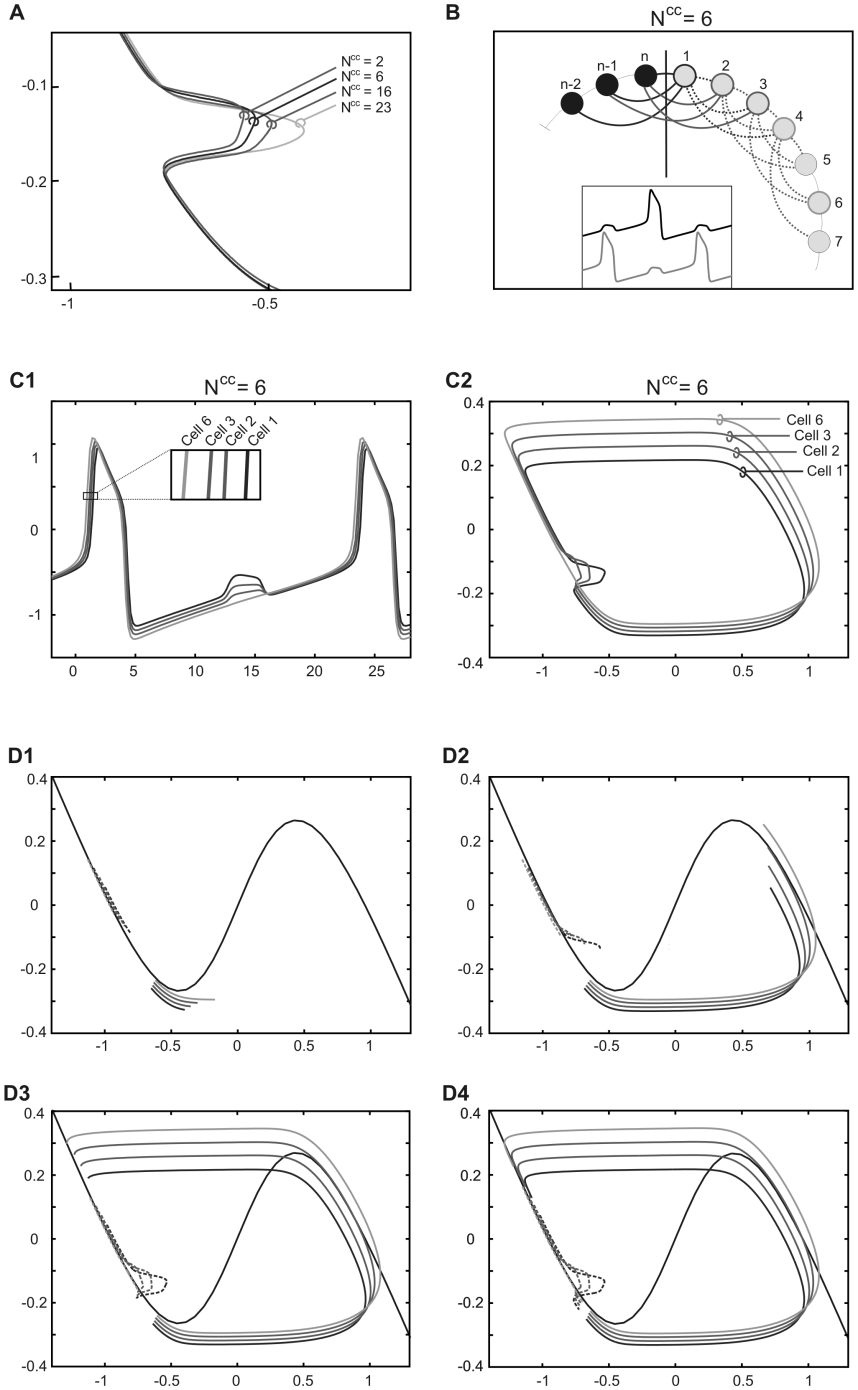
Effect of network connectivity on the trajectory of cells expressing AP pattern. A. Deflection of cells trajectory resulting from interaction with the active cells for different connectivity parameter (N^cc^). B. Wiring diagram of connectivity for N^cc^ = 6. Shown are connections close to the border between two groups of cells (vertical line) (see also voltage traces in insert). C. Voltage traces (C1) and phase plane trajectories (C2) of cells belonging to one of the groups. Shown are same cells as in B. D. Consecutive steps of phase plane evolution (D1-D4) of the two groups of cells (solid and dashed lines). Shown are the same cells as in C and analogous cells from the other group. Parameters: N = 24, g^el^ = 0.22 (A, C-D). Phase plane coordinates: *V* (abscissa), *W* (ordinate) (A, C2, D).

As illustrated in Fig. 5A, decreasing the number of synaptic connections (N^cc^) at a given value of g^el^ diminished the size of the critical deflection. In view of our previous analysis this indicates that at the critical deflection a cell with fewer connections is further away from the knee of the *V*-nullcline than a cell connected to a larger number of cells. Therefore the solution is more robust – the value of g^el^ may be increased much further until the solution has to disappear due to geometry of the nullcline.

We will show now that this increased robustness of the AP solution for small N^cc^ is due to the asynchronous behavior of cells belonging to one of the two antagonistic groups (see grey vs. black voltage traces in the insert, Fig. 5B). For example, for N^cc^ = 6, cells belonging to one group have different voltage traces (Fig. 5C1) and different phase plane trajectories (Fig. 5C2) depending on their position within the group. “Middle group“ cells are not connected at all with the members of the other group (see cells. 4-7, Fig. 5B) and these cells evolve almost like free cells (cf. trajectory of cell 6 in Fig. 5C2 with free cell trajectory, Fig. 4A). By contrast, “edge” cells, situated at the border of the group, make of the most connections with the cells belonging to the other group (see cell 1, Fig. 5B) with the same total coupling strength as in fully connected network. However, the interaction with members of the same group modifies the edge cell's trajectories and decreases the size of the critical deflection in comparison with the fully connected network, in which all cells express identical voltage traces and therefore there is no intra-group interaction (Fig. 5A).

### De-synchronization within groups of cells firing in anti-phase (AP)

In networks with low N^cc^ we also observed de-synchronization of firing within each of two groups of cells expressing AP behavior: cells with less intergroup connectivity jumping ahead of cells more strongly connected with members of the opposite group (see insert, Fig. 5C1 and Fig. 5B). This can be understood by analyzing the cells' phase plane trajectory. Fig. 5D1 illustrates a phase of the cycle when one of the groups evolves in the silent phase just before the jump up to the active phase (see solid color curves) whereas the other group is less advanced in the silent phase (see dashed color curves). As we already mentioned above, in both groups trajectories of “edge” cells (see dark blue and red curves), influenced by larger coupling currents, are farther away from the free nullcline (black curve) than less affected trajectories of cells situated in the middle of the group (see grey and green curves). In particular, in the advanced group the trajectories of “edge” cells are shifted more down by hyperpolarizing currents than trajectories of “middle” cells (cf. dark blue and grey solid curves, respectively, Fig. 5D1). Before a given cell jumps up to the active phase it must reach the knee of its nullcline: this is the slowest part of the cell's trajectory since the speed of evolution along the *V*-nullcline depends on the distance from the *W*-nullcline which is shortest close to the knee (see Figure 4A). The more the nullcline is shifted down by the hyperpolarizing current the more slowly a cell evolves close to the knee. Therefore the “middle group” cells, less hyperpolarized (see grey and green solid lines, Fig. 5D1-2), reach the knee of their nullcline (not shown) and jump up to the active phase before the “edge” cells (see dark blue and red solid lines, Fig. 5D1-2). The cells trajectories differ also in the active phases and here again the “middle” cell trajectories, less compressed by the electrical coupling, approach the free cell trajectory (cf. grey or green solid curve, Fig. 5C2 and black curve, Fig.4A). Consequently, these cells jump down on the left branch of the *V*-nullcline more far away from its knee than “edge” cells, which become “leaders” of the group (cf. grey and dark blue solid lines, Fig. 5D3). During the evolution along the left branch of the *V*-nullcline the distance between “leaders” and “followers” decreases (cf. vertical distance between solid lines after jump down and close to the knee, Fig. 5D3-4) because “followers” are further from the *W*-nullcline and therefore evolve with the larger speed. Finally, during jump up to the active phase, the order of cells is reversed and “followers” become “leaders”, as described above (see Fig. 5D1).

In order to place the results obtained in a more biological context we have used instead of a ring - a chain of coupled cells, as in models of spinal cord CPGs. Interestingly, the results were fairly similar, showing increased robustness (by about 50%) of the AP solution in comparison to fully coupled network and also showing asynchronous behavior of cells within the same group. Here cells with less intergroup coupling fired before the other cells as in the ring model. However, whereas in the latter case the less influenced cells were located in the middle of their group, in the former model they were located at the edge of the chain (data not shown).

### Why patterns containing more phases are less robust

**Figure 6:**
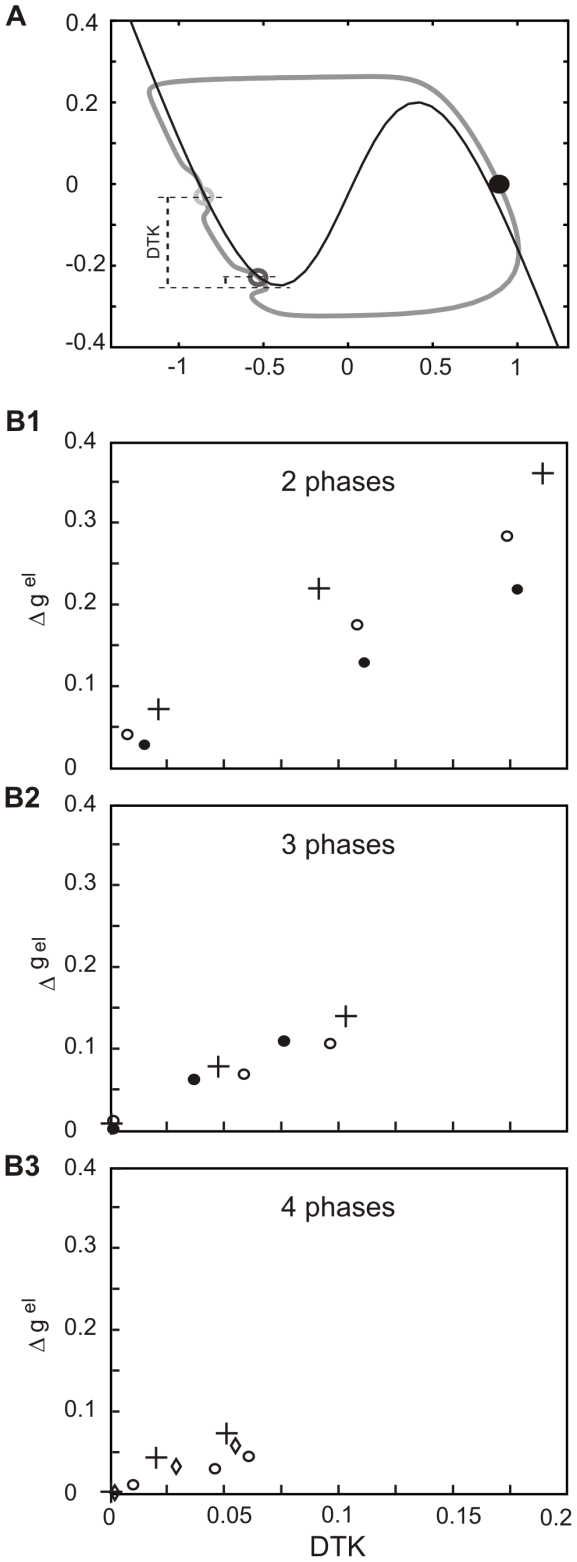
Position of a critical deflection of a trajectory as a function of number of phases in the pattern. A. Phase plane evolution of cells expressing the 3-phase activity pattern. Shown are the cells trajectory (solid grey curve) and cells nullcline (solid black curve). During the active phase of one group of cells (black disc) two other groups (light grey and dark grey circles) evolve at a different distance to the knee (DTK) of the nullcline. A deflection closer to the knee is critical for stability of the pattern. From here cells (dark grey circle) will jump up below the knee and cease the solution if a slight increasing of g^el^ (Δg^el^ = 0.001) shifts the nullcline up. Note that for cells evolving at the upper deflection much larger increment of g_el_ would be necessary to produce a jump. B. A distance Δg^el^ from the given point in (g^el^, N^cc^) space to a value of g^el^ where a transition to synchrony occurs, is plotted against DTK for 2, 3 and 4-phase solutions. For each of N^cc^ = 2, 10, 23 in the 2 and 3-phase solution (see crosses, open circles, full circles, respectively, B1-2) and for N^cc^ = 2, 6, 10 in the 4-phase solution (see crosses, diamonds and open circles, respectively, B3) 3 values of g^el^ were chosen: very small (g^el^ = 0.01), close to the value for which a transition to IP occurs and an intermediate value of g^el^. Note that a range of g^el^, in which a given solution exists, diminishes with increasing number of phases and so does the range of DTK (cf. B1–B3). Only robustness of the 2-phase solution (AP) is dependent on the connectivity pattern (cf. B1 with B2-3). Parameters: N = 24. Phase plane coordinates: *V* (abscissa), *W* (ordinate).

In the next step we asked why increasing the number of phases in the pattern dramatically decreases its robustness, as described above (cf. top with middle and bottom rows in Fig. 3A and B). In order to answer this question we first considered the phase plane trajectory of the 3-phase solution in the fully coupled network (grey curve, Fig. 6A). Here, when one group of cells is active (black disc) the other two groups evolve in the silent phase (see light gray and dark gray circles) along two deflections of the trajectory resulting from a coupling with the active group. Previously, we have shown that the 2-phase solution disappears when such a deflection becomes large enough so the cell can jump directly to the active phase and join the active group (see Fig. 4C2). This occurs when, due to the coupling current, the knee of the nullcline is shifted above the current position of the cell in the phase plane.

Which among the two deflections illustrated in Fig. 6A1 become critical for existence of the 3-phase solution? Obviously, a cell belonging to the delayed group (light grey circle) is farther away from the knee of the nullcline (black curve) than a cell from advanced group (dark grey circle) (cf. Distance To the Knee, DTK). The latter, with a slight increase of g^el^, will find itself below the knee of the new nullcline and will jump directly to the active phase cutting off the existence of the 3-phase solution (not shown). We therefore conclude that for multiple phase solutions, when the trajectory contains several deflections, a deflection closest to the knee becomes critical.

Importantly, DTK of critical deflections (calculated for the moment when active cells cross *W* = 0) decreases with increasing number of phases. This is shown in Fig. 6B1-3, where for arbitrary chosen points in (g^el^, N^cc^) space a distance Δg^el^ from a given point to the border of existence of a given solution (i.e. to a value of g^el^ where a transition to synchrony occurs) is plotted against DTK for 2, 3 and 4-phase solutions. For 2 and 3-phase solutions we chose N^cc^ = 2, 10, 23 (see crosses, open circles, full circles, respectively, Fig. 6B1-2) and for 4-phase solution N^cc^ = 2, 6, 10 (see crosses, diamonds and open circles, respectively, Fig. 6B3). For each of the above N^cc^ values we choose 3 values of g^el^: very small (g^el^ = 0.01), close to the value of g^el^ at which the transition to IP occurs (determined in simulation) and an intermediate value of g^el^. Visibly, DTK is a good indicator of the stability of a solution since it is well correlated with Δg^el^ and becomes very small close to the border of existence of the solution. The range of g^el^, in which a given solution exists, diminishes with increasing number of phases and so does the range of DTK (cf. Fig. 6B1–B3). We can also see that, in agreement with results shown in Fig. 3, only robustness of the 2-phase solution (AP) is dependent on the connectivity pattern (cf. Fig. 6B1 with Fig. 6B2–3).

### Effect of the connectivity on patterns containing more than 2 phases

**Figure 7:**
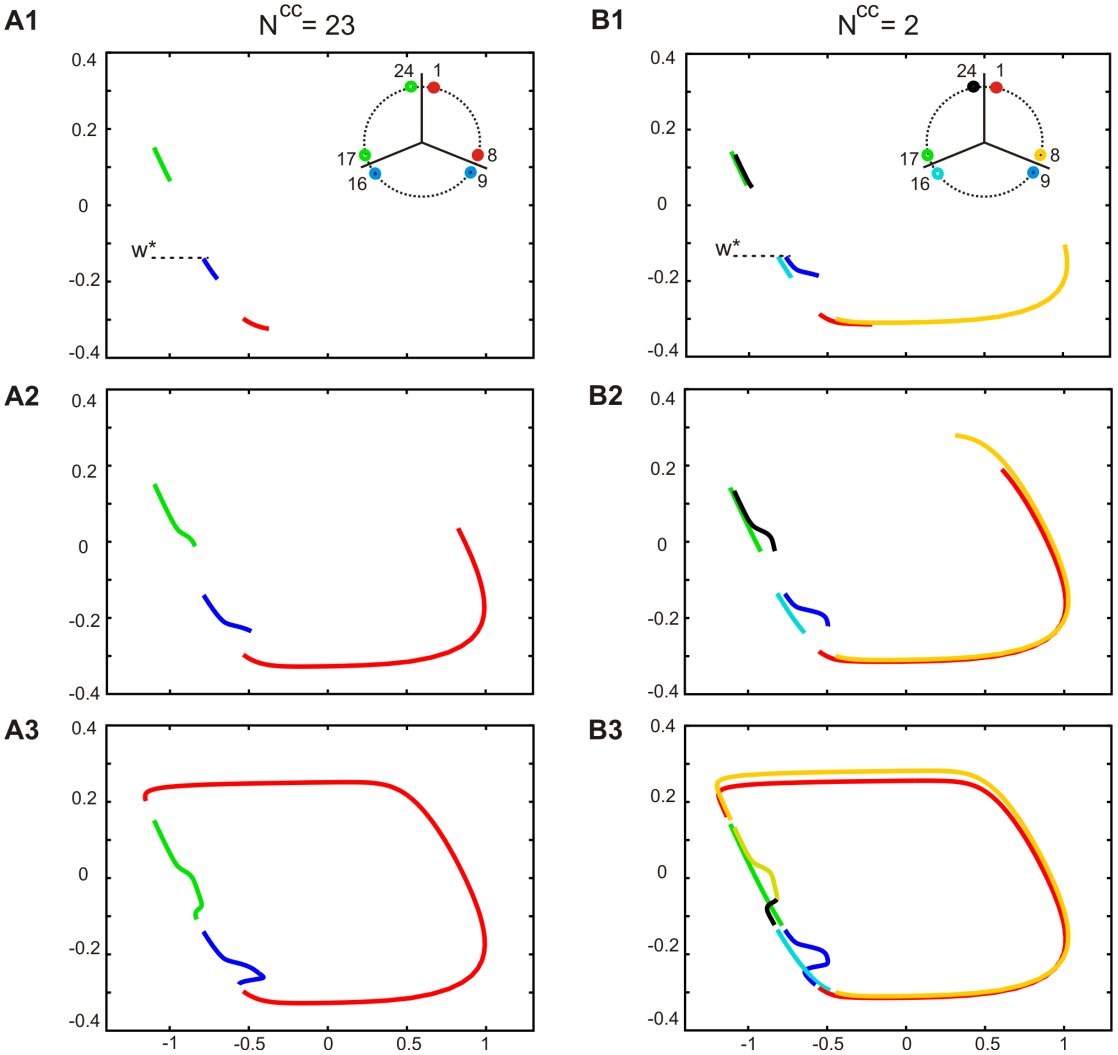
Effect of network connectivity on the trajectory of cells expressing 3-phase pattern. Shown are consecutive steps of phase plane evolution of cells expressing 3-phase activity pattern in fully connected (A) and N^cc^2 network (B). A. Cells belonging to different groups (solid color curves) evolve in the silent phase (A1) and during the active phase of a leading group (A2–3). Within each group trajectories are identical. B. Shown is the evolution of cells placed at the borders between groups (insert, B1) starting from the same W* (horizontal line) and during the same time intervals as in A. Cells connected with a less advanced group (like cell 8 from the leading group connected with cell 9, insert) are leaders in their own group during the active phase (cf. yellow and red curve, B1–2). Notice no clear difference in the size of critical (bottom) deflection in A3 and B3. Parameters: g^el^ = 0.18. Phase plane coordinates: *V* (abscissa), *W* (ordinate).

We next asked why decreasing the density of synapses N^cc^, which enlarges the domain of existence of the 2-phase solution, has almost no effect on robustness of the 3-phase solution (cf. upper and middle row, Fig. 3A and B). To answer this question we compared the trajectory of the 3-phase solution in two extreme cases of connectivity patterns, i.e. in the fully connected and N^cc^2 network, for the same value of electrical coupling (g^el^ = 0.12) (Fig. 7A1–3 and B1–3).

Fig. 7A1 shows the evolution of 3 groups of cells: leading, middle and following (see red, dark blue and grey curves, respectively) during a time interval in the silent phase. In Fig. 7B1 we considered the evolution of 3 analogous groups, starting from the same value of *W* for cell 9 (middle group) (see dark blue curve), during the same time interval. As expected, in the fully connected network, cells belonging to one group evolve identically (Fig. 7A1), whereas in the N^cc^2 network they have different trajectories, as shown in the example of “edge” cells (cf. red (cell 1) and yellow (cell 8), dark blue (cell 9) and light blue (cell 16), grey (cell 17) and green (cell 24) curves, Fig. 7B1). Moreover, “edge” cell 8 jumps up to the active phase before “edge” cell 1 (cf. red and yellow curve, see also insert, Fig. 7B1). This results from the difference in the hyperpolarizing currents: cell 8, connected only with cell 9 (middle group), undergoes a smaller hyperpolarization than cell 1, connected to cell 24 (follower group), due to a smaller distance in *V*. Therefore cell 8 first reaches the knee of its nullcline and jumps up (see yellow curve), which produces depolarization of cell 9 from the middle group (see deflection of dark blue curve). The jump up of cell 8 produces also, via intra-group connectivity, a depolarization of cell 1 (from leading group), which follows cell 8 (see red curve) (Fig. 7B1). With cell 1 active depolarization of cell 24 also occurs (see black curve, Fig. 7B2) and both deflections of trajectories (corresponding to depolarization of cells 9 and 24) last until cells of the leading group jump down (see red and yellow curve, Fig. 7B2–3).

Notice that the size of deflection must be reduced in N^cc^2 network compared to the network with all-to-all coupling due to intra-group connectivity and a gradient of *V* within the group, as it was in the case of the AP pattern (see above). However, in the 3-phase pattern a critical deflection is attenuated also via interaction with cells from the other, less advanced groups, of a smaller *V*. Whereas such interaction between groups is fully present in the all-to-all coupled network decreasing N^cc^ reduces the number of long distance connections and for N_cc_ = 2 the cell which undergoes a critical deflection of the trajectory (here cell 9, dark blue curve, Fig. 7B1-3) is connected only with cell 8 of the active group and cell 10 of its own group (insert, Fig. 7B1).

We therefore conclude that in a solution containing 3 or more phases there are two factors diminishing the critical deflection: interaction with cells less advanced in the silent phase and interaction within the same group of cells. With decreasing the density of synapses in the networks the effect of the former is reduced whereas the effect of the latter is enhanced. By contrast, in the 2-phase solution, where only one group of cells evolves in the silent phase while the other group is active, decreasing N_cc_ has a pure stabilizing effect on the solution via intra-group coupling.

### Effect of cells' duty cycles on the maximal number of phases in a solution

In our simulation we were not able to divide the network, using transient switching stimuli, into more than 4 equally numerous groups of cells expressing different phases in oscillatory cycle i.e., to obtain a solution containing more than 4 phases. We consider now the possibility that such a solution does not exist due to the limited space in which the evolution of our model oscillators was possible in the phase plane.

**Figure 8:**
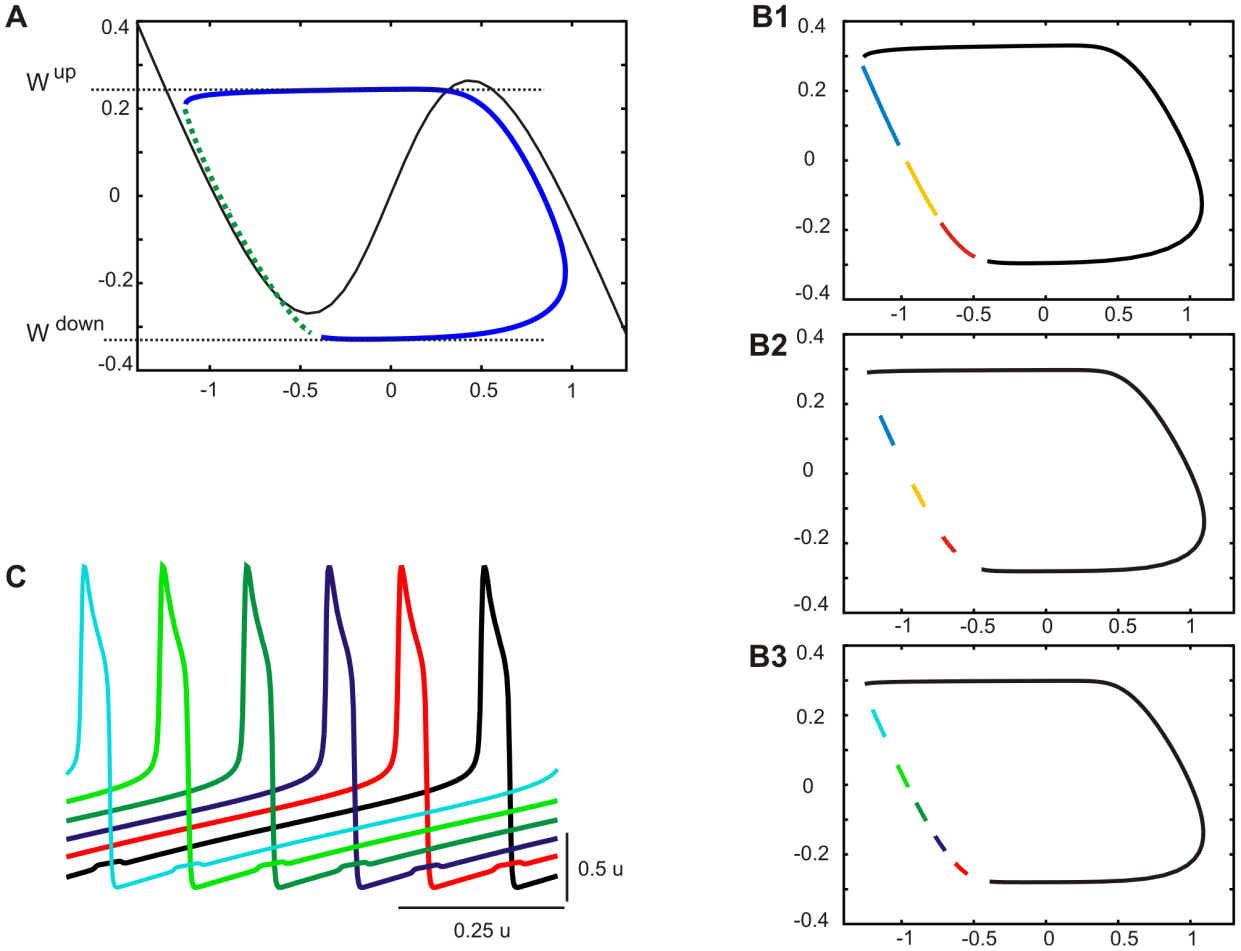
Maximal number of phases in the activity pattern. A. A general shape of a multiphase pattern trajectory. The trajectory (dark blue curve) is placed between limit values of *W* (*W^up^* and *W^down^*) (horizontal lines) at which jumps take place. During the silent phase (dotted green curve) a number of deflection may occur (not shown), equal to number of phases in the pattern - 1. The free nullcline is shown as a reference curve (black solid curve). B. Trajectories of cells belonging to different groups expressing 4-phase pattern (B1-2) or 6-phase pattern (B3) during a full active phase of a single cell. For the 4-phase pattern shown are trajectories of cell 1, 7, 13, 19 (black, red, yellow and blue curves, respectively, B1-2). For the 6-phase pattern shown are trajectories of cell 1, 5, 9, 13, 17, 21, (black, dark blue, green, light green and light blue curves, respectively, B3). Notice the effect of shortening the cells' duty cycle (cf. length of trajectories in the silent phase, see color curves in B1 and B2). C. Voltage traces of cells expressing the 6-phase pattern. Only cells connected with a more advanced group are shown. Parameters: N = 24, N^cc^ = 2, g^el^ = 0.08. Phase plane coordinates: *V* (abscissa), *W* (ordinate). Cells numbers as in Fig. 7B (insert).

Fig. 8A shows the general shape of the trajectory of a multiphase solution containing the active phase (solid dark blue curve) and silent phase where multiple deflections may occur (dotted green curve). As illustrated, the trajectory is situated between two extreme values of *W* (see horizontal dotted lines): the upper (*W^up^*), indicating cells' position just before a transition to the silent phase (jump down), is placed below the right knee of the free nullcline (solid black curve) at the distance determined by the value of hyperpolarizing current resulting from interaction of active cells with cells evolving in the silent phase; the bottom (*W^down^*), placed below the left knee of the free nullcline, indicates the position of cells just before a transition to the active phase (jump up), when they are under the inhibitory influence of cells less advanced in the evolution in the silent phase. How many cells may evolve simultaneously in the silent phase in between these two limit values *W^up^* and *W^down^*? Fig. 8B1 illustrates the evolution of cells expressing the 4-phase pattern (to simplify the figure only one cell of each group is shown). Assuming that during τt a cell from a leading group jumps up (starting from *W^down^*), evolves in the active phase and jumps down (at *W^up^*) (black curve) whereas at the same time cells belonging to the remaining groups evolve in the silent phase (see red, black and blue curve). In the next part of the cycle all 4 groups will evolve together in the silent phase until each cell reaches a starting position of a more advanced cell whereas the most advanced cell (red curve) will reach *W^down^* and the next jump up will occur (not shown). Thereby ¼ of the cycle will be completed. If a solution containing more than 4 phases is to exist we need a space (distance in *W*) for at least one more cell evolving during Δt in the silent phase, equivalent to the length of any of the trajectories expressed by 3 groups (see red, black and blue curves, Fig 8B1). Obviously, in the presented example such space is not available (compare space between trajectories of different groups with their length). In other words, in our example a time interval between the offset of the active phase (jump down) and the onset of the next active phase (jump up), corresponding to time of evolution of all groups in the silent phase, will be smaller than the duration of the active phase:

¼ T−Δt<Δt

so a solution containing more than 4 phases will not exist. However, decreasing a cells duty cycle, i.e. Δt/T, assures that ¼ T−Δt>Δt, as illustrated in Fig. 8B2 (compare length of trajectories with intervals between them) and thereby assures the existence of a solution with a larger number of phases (see phase plane evolution, Fig. 8B3 and voltage traces of cells expressing the 6-phase solution, Fig. 8C).

### Asymmetrical 2-phase solutions

**Figure 9:**
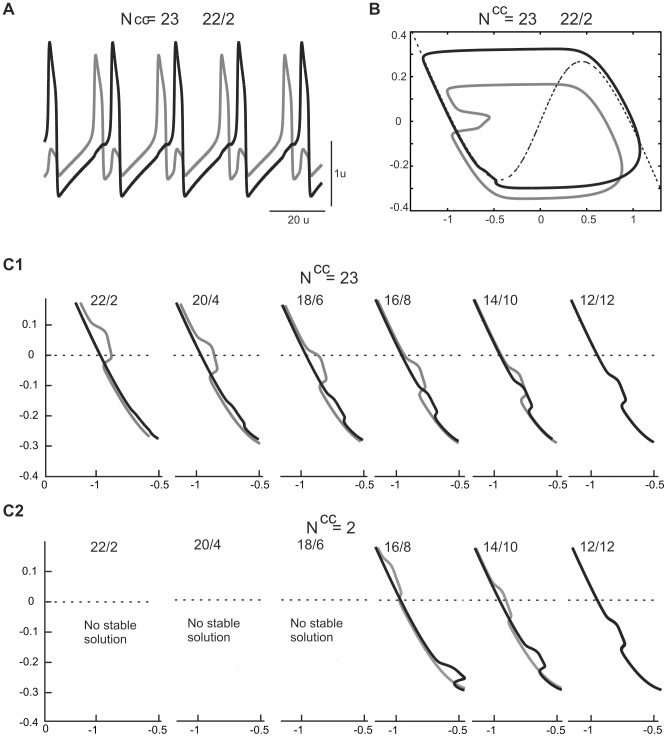
Asymmetrical 2-phase activity pattern. A. Voltage traces of 2 groups of cells expressing asymmetrical behavior in a fully connected network. Leading is the group of a larger size (22 cells) (black solid curve), the following group of a smaller size (2 cells) (grey solid curve) expresses activity of a diminished amplitude (A). B. Phase plane trajectories of the same groups as in A. C. Phase plane position of deflections of trajectories of both groups in a function of group size ratio in fully coupled (C1) and N^cc^2 networks (C2). Parameters: N = 24, g^el^ = 0.06. Phase plane coordinates: *V* (abscissa), *W* (ordinate) (B, C).

So far we only analyzed patterns in which multiple groups of cells expressed activity in different phases of the cycle, with equal group sizes. In particular, in the 2-phase pattern two identical groups of cells were oscillating in anti-phase. Figure 9A shows the activity pattern expressed in the fully connected network by 2 groups of cells of different size (here containing 2 and 22 cells). As illustrated, cells voltage traces are different: the larger group (LG; black curve) expresses oscillation of a higher amplitude in the active phase but lower amplitude of a deflection occurring during the silent phase than the smaller group (SG, grey curve), which follows LG with a small phase lag.

Asymmetry is present also in the phase plane trajectories. As illustrated in Fig. 9B, the trajectory of SG (grey curve) is situated more far away from the free nullcline (dashed curve) and is thereby more affected by the coupling current than the trajectory of LG (black curve). This is due to the unequal number of intergroup connections made by a single cell belonging to SG and LG: the intergroup coupling is stronger in SG (each cell makes 22 synapses on LG and 1 synapse on SG) than in LG (each cell makes 1 synapse on SG and 22 on LG). Asymmetry in the coupling strength results also in the difference in the size and position of deflections occurring during the silent phase of the two groups (Fig. 9B), which reflect the fact that SG is a leader whereas LG is a follower in the cycle (cf. active phase of LG occurring soon after jump down of SG, Fig. 9A). Indeed, with diminishing asymmetry between the size of the two groups two deflections approach each other (see grey and black curves), their *V*-amplitudes become similar and finally, for equal group sizes, they fuse in a single deflection, reflecting symmetrical evolution of the two groups in the anti-phase solution (Fig. 9C1).

Note that increasing the asymmetry between the groups decreases the size of the deflection occurring in the trajectory of LG and shifts it down (black curve, Fig. 9C1). As explained previously, the robustness of a multiphase solution depends critically on the size and position of the deflection occurring in the silent phase of the phase plane trajectory. This further suggests that the robustness of the 2 phase asymmetrical solution may be similar in the case of weak asymmetry, when the deflection is large and far away from the knee and in the case of stronger asymmetry between the two groups when small deflection is situated close the knee of the nullcline.

**Figure 10:**
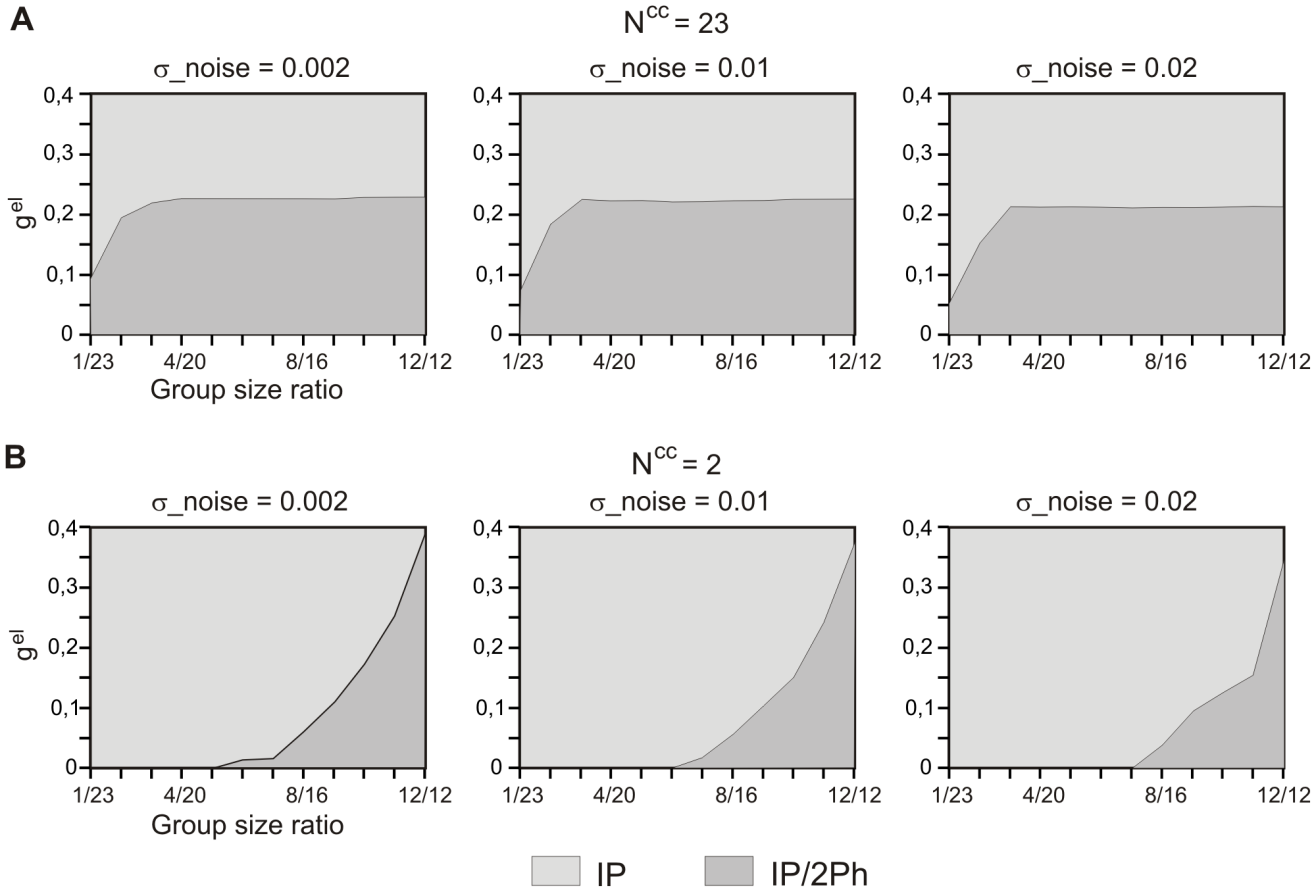
Occurrences of asymmetrical 2-phase patterns as a function of electrical conductance and group size ratio. Domains of stability of asymmetrical 2-phase behaviors in fully coupled (A) and N^cc^2 networks (B) for different levels of noise are shown. Whereas in fully coupled network almost all asymmetrical network divisions are equally stable (A) the ring connectivity strongly promotes symmetrical patterns (B). Parameters: N = 24.

Indeed, as illustrated in Fig. 10A, where domains of stability of the 2-phase solution with different group size ratios are shown for different amplitudes of noise, robustness of the 2-phase solution in the fully coupled network is almost independent of asymmetry between the 2 groups of cells. (Note that range of g^el^ where the solution for 12/12 group size ratio is stable is identical to the domain of stability of the 2-phase solution for N^cc^ = 23 in Fig. 3A, where only symmetrical solutions are considered.) In contrast, in the N^cc^2 network the 2-phase solution exists only for relatively similar groups size and disappears with stronger group size asymmetry (see Fig. 10B) (here again 12/12 group size ratio corresponds to N^cc^ = 2 in Fig. 3A). This is due to the fact that for low N^cc^ asymmetry in the size of the two groups does not result in asymmetry in the coupling between members of the groups, as for the fully coupled network. For example for N^cc^ = 2, the only two cells in each group which are directly coupled with the other group (edge cells, see Fig. 5B) are coupled with a same conductance strength equal to g^el^/2, i.e, with a coupling strength which was present in a symmetrical group division. This is further illustrated in Fig. 9C2 where deflections are shown occurring on the trajectories of edge cells in two groups as a function of asymmetry between the two groups. Here, with increasing asymmetry, a deflection of trajectory in LG is shifted down (see black curve), as for all-to-all coupling, but still with a relatively large amplitude (cf. Fig. 9C2 and C1) rapidly approaching a position where the 2-phase solution disappears.

In summary, two groups of cells of unequal size may express the 2-phase activity pattern in which the active phase of a larger group is expressed first and followed after some phase lag by the active phase of the smaller group. The larger the asymmetry between the size of the two groups, the smaller the phase lag between the leader and follower. Robustness of the solution in the fully coupled network is relatively independent of the difference between two group sizes whereas in a network with a small number of connections the solution only exists for a low level of asymmetry.

## Discussion

Obviously, if cells are only coupled electrically, then IP phase-locking exists and is stable for all values of electrical coupling. This has been shown for both relaxation oscillator and integrate-and-fire neuron models, although the synchronization mechanisms are quite different [22]–[28]. Previous studies have also shown that weak electrical coupling may lead to AP locking in networks composed of different neuronal model cells [22]–[28], [31]. In the present study we are going along this line of research, since our results demonstrate, for the first time, a much richer variability of multiphase activity patterns that may be expressed by a network comprised of electrically coupled relaxation oscillators.

Indeed, we have shown that a large-scale network of oscillatory cells with short duty cycles, coupled solely by electrical synapses, may express multiple stable activity patterns, both symmetrical and asymmetrical. Among symmetrical patterns, in which equal numbers of cells fire at different phases of the oscillatory cycle, we found 2, 3 and 4-phase patterns coexisting with the 1-phase pattern, i.e. with synchronous or in-phase (IP) behavior (Figures 1–2). Also patterns containing more than 4 phases were possible to obtain by diminishing the cells duty cycle (Fig. 8). Such multiphase activity patterns were stable over a range of electrical conductance (g^el^), which was narrower the larger the number of phases expressed in a given behavior (Fig. 3).

Looking for the effect of network topology (Fig. 1A) on the robustness of expressed patterns we found that only the existence of the 2-phase behavior was strongly affected. Indeed, the robustness of the anti-phase pattern (AP) increased when network connectivity approached a pure ring organization (N^cc^ low) (Fig. 3). On the other hand, among 2-phase asymmetrical patterns, i.e. patterns of different number of cells contributing to each phase, no stable solution existed in a ring network if asymmetry between two group sizes was high (Fig. 10B). In contrast, in the fully coupled network the stability of 2-phase patterns was fairly independent on the two group size ratios (Fig. 10A). Therefore, the main effect of the ring topology on the network is to eliminate asymmetrical 2-phase patterns and promote AP behavior. Moreover, such network architecture gives rise to a hierarchical organization of activity: during each of the main phases of the activity pattern a group of participating cells do not fire synchronously but in a given sequence determined by the cells' interconnections.

Since a rigorous mathematical explanation of a given dynamic behavior is generally neither easy to achieve nor easy to understand for a reader without mathematical background, we have provided a simplified explanation of our main findings described above using a geometrical analysis of phase plane evolution.

First, we explained that a deflection of the phase plane trajectory occurring due to interaction between antagonist groups of cells during the cell's evolution in the silent phase is critical for the existence of the AP solution (Fig. 4). Second, we showed that the robustness of the AP solution increases in the ring network due to interaction via electrical coupling within the agonist group of cells which reduces the size of the critical deflection (Fig. 5). Third, we explained that solutions containing larger numbers of phases are less robust due to the position of the critical deflection in the phase plane, shifted toward the knee of the nullcline (Fig. 6B). Fourth, we showed that in the case of patterns containing more than 2 phases the impact of network topology on the stability of a solution is limited due to the opposite effect of inter- and intra- group coupling currents influencing the size and position of a critical deflection (Fig. 7). Fifth, we demonstrated that when one of the groups of cells contributing to a given activity pattern is active, the remaining groups evolving in the silent phase have a limited space available in the phase plane. This determines how large the variety of patterns that may be expressed by the network is for a given electrical coupling strength: the shorter the cell's duty cycle the larger the maximal number of phases in the pattern (Fig. 8). Finally, we explained how asymmetry between the sizes of the two groups expressing 2-phase behavior influences the phase plane trajectory of cells for different connectivity patterns. We showed that highly asymmetrical 2-phase patterns cannot exist in a network with a small number of connections per cell due to the size and position of the critical deflection (Fig. 9 and 10).

Our model network is assembled from neurons arranged in a virtual ring, with a connectivity parameterized by a number of connections of a single cell to its closest neighbors. Although such ring network architecture has so far never been described in biological systems this is a convenient way to control the local connectivity in a model of gap junction coupling between neighboring cells [32]. Indeed such network organization allows edge effects to be avoided which would be present, for example, in a line of coupled oscillators. In order to place the results obtained in a more biological context we have used instead of a ring - a chain of coupled cells, as in models of spinal cord CPGs, and found similar increase of the robustness of the AP pattern as compared to the fully coupled network.

It has been shown in the previous studies of the dynamics of electrically coupled oscillatory cells that decreasing the cells duty cycle (or frequency of firing) assures stability of the AP pattern [22]–[24]. Interestingly, our results indicate that it also reveals other possible oscillatory behaviors of a more complex structure. What may be the physiological function of such oscillations?

Although it is difficult to perform research work on the physiological role of gap junctions during early development of the CNS, there are some studies that indicate that electrical coupling between neuronal cells may play an important role in the formation of local connectivity [33]–[37]. Moreover, it has been reported that the transient electrical coupling may play a crucial role for the establishment of chemical synapses [38]–[40]. More recently, it has been demonstrated that transient electrical coupling between radially aligned sister excitatory neurons regulates the subsequent formation of chemical synapses within the group of cells that was electrically connected [41]. One important issue in all these studies is the capability of neurons to “sense” each other in order to be able to form functional clusters. However, the classic views on the electrical synapses function is their ability to equalize the membrane potential between connected cells so that the group of neurons will fire in synchrony [42]. Here, a paradoxical effect of the gap junction communication is that if the potentials of cells are identical, the current flow via such synapses is equal to zero and therefore there is no information exchange between the neuronal cells, at least no transfer of charged particles. Using a modeling approach we were able to show that neurons solely electrically coupled, if the number of synapses per cell is small (in ring-like or chain organization), may express within a given cluster a small phase shift allowing a given cell to “sense” the other cell partners via gap junctions during a small time interval within each oscillatory cycle. One can imagine that such a mechanism may play an important role in early development to create clusters of cells where subsequent chemical synapses will take place.

Another possible function of such patterned activity in a solely electrically coupled network is the possibility to create early in development some proto networks expressing complex patterns of activity. However, these patterns are quite unstable: they always coexist with IP pattern and other multiphase patterns and can switch from one to the other configuration. We have previously shown that such switching exists and that transient stochastic or sensory signals may produce transitions between IP and AP behaviors [43], [44]. Interestingly however, introducing chemical inhibition should have a stabilizing effect on multiphase patterns. Indeed, the action of inhibitory chemical synapses, which will take place only during the active phase of cells, will diminish the size of the deflection of cells trajectories occurring during electrical interaction between active and silent groups which, as we have shown in this study, is critical for the stability of a pattern. Therefore one can postulate that during early development a population of electrically coupled neurons may generate multiphase spatiotemporal patterns that provide a way to organize the development of chemical synapses within such proto-networks. In turn, the establishment of chemical synapses will stabilize generated patterns and the network will become more robust in the face of incoming information.

In our previous study on the role of electrical synapses in developing networks, we have shown a potentiality of gap junctions to equalize expression of multiphase activity of premature inhibitory networks into one embryonic pattern [43]. Here quite the reverse possibility is shown i.e., that a variety of premature multiphase patterns of activity can be expressed in populations of neurons coupled solely electrically, where inhibition will be established in the future.

## Materials and Methods

### The Cell Model

As previously described in [31] cells in the network are modeled by a set of first order differential equations, each cell contributing two state variables to the set: the instantaneous membrane potential (*V_i_*) and a slow recovery current (*W_i_*), dependent on the membrane potential. The variables have the (non-dimensionalised) dynamics defined by equations 1–4.
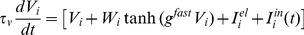
(1)

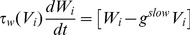
(2)


In equations 1–2 *g^fast^* determines the degree to which the instantaneous voltage-dependent current is N-shaped whereas *g^slow^* models the voltage-dependent activation function of the slow current. Gap junction current is given by 
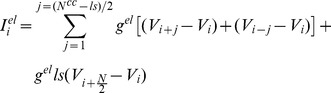
(3)where *g^el^* is a total electrical conductance per single cell and N^cc^ is the number of synapses per cell, *ls = 1* if *N^cc^>N-2* and *0* elsewhere. Here, since the network is organized as a ring of *N* cells with a cell's number increasing in one direction therefore if *i+j>N* then *V_i+j_ = V_N-i-j_* and if *i-j<0* then *V_i-j_ = V_N+i-j_*. Since *N* is a paired number then for the all-to-all coupling (*N^cc^ = N-1*) we add one connection (*ls = 1*). 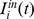 is externally injected input current. ττ and τ_w_ (*V_i_*) are the membrane time constant and the time constant of slow current dynamics, the latter depending on the membrane potential *V_i_*:
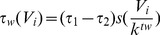
(4)with ττ and ττ specifying the minimum and maximum time constants and thereby determining the durations of the active and silent phases of the oscillator – and *k^tw^* quantifying the rate of voltage dependence. Sigmoidal function *s(x)* is defined as *s(x) = 1/(1+e^x^)*. The complete model defined by equations 1–4 has, therefore, three time constants, two membrane, one junction conductance and one rate constants (the k parameter). For tractability in the current work following parameters are identical for all junctions and neurons respectively, with following values: *g^fast^* = 2, *g^slow^* = 2, ττ = 5, ττ = 50, *k^tw^* = 0.2, ττ = 0. 16.

The parameters have been chosen to model neurons of relatively short duty cycle (i.e. with short fraction of the cycle when the cell is depolarized above threshold and may exert synaptic action). With this choice of parameters, equations 1–4 may be considered as a model of spiking neurons. In the study presented here, the only parameters varied from the defaults is the time constant ττin the example presented in Fig. 8B2-3 and C and the conductance g^el^, except of a network of reciprocal excitatory instead of electrical connections considered in Fig. 4B (see Results). Here we replace 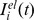
in equation (1) by 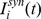 given by
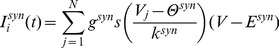
(5)Where and *g^syn^* = 0.05 is the maximal synaptic conductance, *V_j_* is the membrane potential of the presynaptic cell *j*, *E^syn^* = -4 is the synaptic reversal potential whereas *Θ^syn^* = 0 and *k^syn^* = 0.02 are, respectively, the midpoint and steepness of the synaptic activation function.

The model has been implemented as a set of Matlab functions which compute the quantities defined by the equations above and integrate the set of ordinary differential equations using Matlab's standard ode45 solver with the default tolerance parameter settings. The external input currents 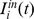 are assumed to be piecewise constant. The implementation has been independently realized using the xpp tool (http://www.pitt.edu/~phase/) and found to give identical results. The model codes are placed in http://ibib.waw.pl/download/MPA/MultiphasePatternAnalysis.zip.

### Analysis Methods

For a given choice of parameters, the network exhibits a number of oscillatory behaviors. In this study, we vary the following parameters: electrical conductance g^el^ and number of synapses per cell (N^cc^), over the range in which interesting behaviors occur, for networks comprising 24 and 60 cells. The reported results generated as follows. For each value of N^cc^ investigated:

1. The model is integrated with time step of 0.2 unit (the model is dimensionless) from initial conditions identical for all cells and for electrical coupling very low (generally *g^el^* = 0.02) so that the IP behavior is generated.
2. Thereafter an attempt is made to switch the network to another oscillatory behavior: some cells receive a 1 unit positive current injection and some a negative current injection of 1 unit amplitude for 0.2 time units, applied once or in successive attempts, depending on the number of desired phases in the target activity pattern.
3. In the next step we verify whether the obtained solution (see p.2) is stable in the presence of noise. For this aim we apply random current inputs during 250 time units. Random current input is constructed using independent identically Gaussian distributed random values with zero mean and standard deviation σ_noise = 0.002, 0.01 or 0.02 for each 0.2 time unit step. The behavior is then classified for each consecutive increment of g^el^ using algorithms described in [31], [44], [45].
